# Annotations

**Published:** 1894-01-06

**Authors:** 


### ANNOTATIONS.

### A Medical Confessional.

MR. A. H. F. ROUTH, Surgeon to the Bridgewater Infirmary, has made an interesting communication to a medical contemporary, and at the same time set an example of refreshing frankness to the whole profession. Mr. Routh describes the case of a patient suffering from locomoter ataxia, whom he treated by means of Brown-Sequard's testicular fluid. According to the published notes of the case, the temperature and pulse were both quite normal when treatment commenced, the former being 98·6 deg., and the latter 80. After the first injection the pulse dropped to 52, and the temperature to 97 deg. After the second the pulse fell to 36, and the temperature to 95 deg. The treatment was continued for four weeks, three injections being given each week. Throughout the time the pulse remained at or near 36, and the temperature at or near 95 deg. At the close there were no manifestations of improvement of any kind, and the patient expressed himself as feeling no better in any respect. Mr. Routh says that he seemed to be weaker at the end of the month than at the beginning, and when one considers the profound physiological depression indicated by the temperature and the pulse, one cannot refrain from the speculation that "weaker" is somewhat too "weak" a word to describe the actual condition. Mr. Routh is worthy of all honour for his frankness; but whoever is familiar with the pathology and history of locomoter ataxia, and of the composition of Brown-Sequard's fluids, will be disposed to pronounce the whole experiment a little ridiculous. It is surely time that this aspect of Brown-Sequardism was played out.

### "Rights" in Hospitals.

THE monthly journal, *Forward*, of the Birmingham Hospital Saturday Fund, discussed in a recent number the question of the "right" to hospital treatment of subscribers to the Saturday and workshop collections. There is no such "right" says *Forward*. "Not until a man pays the full market value of the medical treatment and medicine which he receives can he claim these things as rights." It will not be pretended that a penny a week subscribed in a workshop, or a few coppers dropped into the collecting box on Hospital Saturday represents the "full market value" of the treatment and medicine accorded to the average workman by Birmingham hospitals in a twelvemonth. It is somewhat of a surprise to us that even a workman should suppose that such small advances in money could establish such large returns in rights. The workman is a man, not a child; and he knows that sixpence at the grocer's or butcher's can purchase sixpence worth of goods—and no more. Why then does he imagine that at hospitals sixpence can purchase from ten shillings to fifty pounds worth of medical treatment, medicine, and food? He probably thinks that because the hospital is a "charity" he has a right to its resources. But when a subscriber gives money to a charity he does not pay a debt, he gives his money for charitable purposes, and the recipient of the benefits of the charity receives those benefits as charities, not as rights. But, argues a workman, "look at the handsome total we raised last year—not less than £6,000—does that give us no rights?" The answer is obvious. Probably 60,000 persons, or more, joined in raising the £6,000, and the cost of doctoring the 60,000 for a year was not £6,000, but fifteen or twenty times that amount. Does the grocer, because a thousand workmen pay him £500 a week, allow any one particular workman to claim a "right" to purchase a shilling's worth of soap for a halfpenny? This whole claim of "rights" in hospitals, by virtue of annual subscriptions, is absurd. Hospitals are charities. Subscriptions to them are free gifts for charitable purposes, and the only possible "right" a subscriber can have is the right of insisting that the money he subscribes shall be used for such particular charitable purposes as the charity professes to carry out.

### Bacillus Prodigiosus in the Pantry.

Last July, Dr. Klein was asked to visit the larder of a certain mercantile house, and to report upon some curious "pink spots" on roast beef and boiled fish, which had attracted the attention of the firm's housekeeper, and which had also been noticed by the "young men" of the establishment, who were expected to eat the beef and fish. One joint of roast beef, which had been served up for a meal, showed a considerable number of "pink patches" on the cut surface, and these extended for some distance into the intermuscular interstices of the joint. A piece of boiled fish also displayed several bright pink patches on the surface. Dr. Klein, as is the manner of bacteriologists, set to work to "cultivate" his new "pink" acquaintances; and after an incubation of forty-eight hours on gelatine, agar, and potato, he had the satisfaction of securing several fine pink colonies of what proved on microscopic examination to be the familiar bacterium, *bacillus prodigiosus*. Two or three questions suggested themselves. First, were the bacilli dangerous? Second, whence had they come? Thirdly, how were they to be got rid of? With regard to the first point, the bacilli had not proved injurious to anybody, because, although several of the young men had frequently eaten the meat, pink patch and all, none of them had suffered in the slightest degree. Secondly, as to the place of origin. Immediately under the windows of the pantry was a disused burying ground. For years this ground had lain undisturbed, but in July last, at the time of the "pink patch" scare, workmen had opened the ground, and made clouds of dust, and with them aerobic microbes, fly in all directions. How, in the third place, were the bacili to be got rid of? For though a particular bacillus may be harmless, he belongs to a family which is, to say the least, "suspect"; and, as yet, we are all disposed to hold that "his room is better than his company." Thorough cleaning of the larder was Dr. Klein's recipe. Hot water, and plenty of it, on floors, walls, shelves, and utensils; for *bacillus prodigiosus* is killed in a few seconds by water at 75 degs. C. The "pink patches" themselves, whenever they appeared in fresh places, were ordered to be carefully cut off and destroyed. This remedy proved effectual, and *bacillus prodigiosus* was banished from the pantry. There is one moral here which we may as well point. It is that bacilli, when noticed early and faced courageously may sometimes be got rid of before they do any harm.

